# Supplementary material for: HIVprotI: an integrated web based platform for prediction and design of HIV proteins inhibitors
Source: J Cheminform. 2018 Mar 9;10:12. doi: 10.1186/s13321-018-0266-y (PMC5845081; doi:10.1186/s13321-018-0266-y)
Supplement: Supplementary file 2 — Additional file 2. Source code of HIVProtI web server. [file 13321_2018_266_MOESM2_ESM.zip › HIVprotI_Source-code/hivprotI/weka-3-6-6/doc/weka/clusterers/UpdateableClusterer.html]

UpdateableClusterer


---


|  |  |  |
| --- | --- | --- |
| **Overview** \| **Package** \| **Class** \| **Tree** \| **Deprecated** \| **Index** \| **Help** \| **Weka's home** | |  |
| **PREV CLASS**   **NEXT CLASS** | **FRAMES**    **NO FRAMES**     **All Classes** |  |
| SUMMARY: NESTED \| FIELD \| CONSTR \| METHOD | DETAIL: FIELD \| CONSTR \| METHOD |  |


---


## weka.clusterers Interface UpdateableClusterer

**All Known Implementing Classes:**: Cobweb

---

``` public interface UpdateableClusterer ```

Interface to incremental cluster models that can learn using one instance
at a time.

**Version:**
:   $Revision: 1.2 $

**Author:**
:   FracPete (fracpete at waikato dot ac dot nz)

---

| **Method Summary** | |
| --- | --- |
| `void` | `updateClusterer(Instance newInstance)`             Adds an instance to the clusterer. |
| `void` | `updateFinished()`             Singals the end of the updating. |

**Method Detail**

### updateClusterer

```
void updateClusterer(Instance newInstance)
                     throws java.lang.Exception
```

:   Adds an instance to the clusterer.

    :   **Parameters:**: `newInstance` - the instance to be added **Throws:**: `java.lang.Exception` - if something goes wrong

---


### updateFinished

```
void updateFinished()
```

:   Singals the end of the updating.


---


|  |  |  |
| --- | --- | --- |
| **Overview** \| **Package** \| **Class** \| **Tree** \| **Deprecated** \| **Index** \| **Help** \| **Weka's home** | |  |
| **PREV CLASS**   **NEXT CLASS** | **FRAMES**    **NO FRAMES**     **All Classes** |  |
| SUMMARY: NESTED \| FIELD \| CONSTR \| METHOD | DETAIL: FIELD \| CONSTR \| METHOD |  |


---
